# Remotely supervised at-home delivery of taVNS for autism spectrum disorder: feasibility and initial efficacy

**DOI:** 10.3389/fpsyt.2023.1238328

**Published:** 2023-09-28

**Authors:** Benjamin Black, Samantha Hunter, Hannah Cottrell, Roee Dar, Nicole Takahashi, Bradley J. Ferguson, Yishai Valter, Eric Porges, Abhishek Datta, David Q. Beversdorf

**Affiliations:** ^1^Department of Pediatrics, Thompson Center for Autism and Neurodevelopment, University of Missouri, Columbia, MO, United States; ^2^School of Medicine, University of Missouri, Columbia, MO, United States; ^3^Department of Neurology, Thompson Center for Autism and Neurodevelopment, University of Missouri, Columbia, MO, United States; ^4^Research and Development, Soterix Medical, Woodbridge, NJ, United States; ^5^Center for Cognitive Aging and Memory, McKnight Brain Institute, University of Florida, Gainesville, FL, United States; ^6^Brain Rehabilitation Research Center, Malcom Randall VAMC, Gainesville, FL, United States; ^7^Department of Clinical and Health Psychology, College of Public Health and Health Professions, University of Florida, Gainesville, FL, United States; ^8^Department of Biomedical Engineering, City College of New York, New York, NY, United States; ^9^Department of Radiology, Neurology, and Psychological Sciences, and the Thompson Center for Autism and Neurodevelopment, University of Missouri-Columbia, Columbia, MO, United States

**Keywords:** autism spectrum disorder, tVNS, taVNS, vagus nerve stimulation, neuromodulation, clinical trial, home trial

## Abstract

**Background:**

Transcutaneous auricular vagus nerve stimulation (taVNS) has potential clinical application for autism spectrum disorder (ASD). At-home sessions are necessary to allow delivery of repeated sessions, and remove burden on patients for daily visits, and reduce costs of clinic delivery. Our objective was to validate a protocol for remote supervised administration for home delivery of taVNS using specially designed equipment and platform.

**Methods:**

An open-label design was followed involving administration by caretakers to 12 patients with ASD (ages:7–16). Daily 1-h sessions over 2 weeks were administered under remote supervision. The primary outcome was feasibility, which was assessed by completion rate, stimulation tolerability, and confirmation of programmed stimulation delivery. The secondary measures were initial efficacy assessed by Childhood Anxiety Sensitivity Index-Revised (CASI-R), Parent Rated Anxiety Scale for Youth with ASD (PRAS-ASD), and Clinician Global Impression (CGI) scales. Sleep measures were also tracked using Cleveland Adolescent Sleep Questionnaire (CASQ).

**Results:**

Across 132 sessions, we obtained an 88.5% completion rate. A total of 22 expected adverse events were reported with headache being the most common followed by transient pain, itchiness, and stinging at the electrode site. One subject dropped out of the study unrelated to the stimulation or the study. Average scores of anxiety (CASI-R, PRAS-ASD, and CGI) and sleepiness (CASQ) were all improved at the 2 week time point. While not powered to determine efficacy, benefits were suggested in this open label pilot.

**Conclusion:**

Remotely supervised, proxy-administered, at-home delivery of taVNS is feasible in patients with ASD. Initial efficacy supports pursuing larger scale trials.

## 1. Introduction

A primary goal in treating autism spectrum disorder (ASD) is to maximize functional independence and quality of life by minimizing the impact of core features and cognitive impairments as well as the effects of co-occurring conditions (1). As of 2013, the prevalence of ASD has rapidly increased to over 2% and has become a significant public health concern (2). Early intervention with behavioral therapy is considered critical, but fewer options are available later in life, and treatment of co-occurring conditions is challenging across the lifespan in ASD. Pharmacotherapy is often a significant component of treatment, primarily directed at psychiatric symptoms that commonly occur in ASD, such as agitation, anxiety, repetitive and obsessive behaviors, and depression (1). However, response to these medications for these psychiatric symptoms are known to be unpredictable in those with ASD (3). Among psychiatric symptoms, anxiety is one of the most prevalent psychiatric symptoms in ASD, with an incidence as high as 50% by age 30 (4), which detracts from the quality of life (5). Thus, an effective treatment approach to reduce anxiety symptoms in patients with ASD would improve quality of life.

Recent evidence has supported the potential of targeting sympathetic and parasympathetic nervous system activity as an approach to the treatment of ASD. Initial work in this area has targeted social and language outcomes. Both social and language benefits have been reported in a small case series of patients with ASD with propranolol, a beta-adrenergic antagonist with anxiolytic properties (6). Early single dose psychopharmacological challenge studies suggest that there may be a benefit in verbal problem solving in ASD (7, 8). as well as in verbal fluency (9). Subsequent larger single dose propranolol challenge studies revealed a significant increase in social functioning as indicated by scores on the Conversational Reciprocity component of the General Social Outcomes Measure (GSOM) (10). Recently, a double-blind placebo controlled trail was performed examining the effect of propranolol in ASD. While improvements in social functioning and on verbal tasks were not revealed, a significant impact was found for anxiety (11). Since there is limited evidence for treatment approaches targeting anxiety in ASD, it would be of significant interest in development of novel treatment approaches for this purpose.

Non-invasive electrical stimulation of a critical component of the parasympathetic nervous system, the vagus nerve or transcutaneous vagus nerve stimulation (tVNS) has been previously proposed as a potential therapeutic method for the treatment of ASD (12, 13). By targeting the parasympathetic nervous system, this impacts sympathetic/parasympathetic balance in a nonpharmacological manner. Application involves a battery-powered portable electrical stimulation device with electrodes held on the ear or neck regions. Accordingly, tVNS can be further classified into two categories, based on the aforementioned intended target: (1) delivery to the cervical branch (transcutaneous cervical vagus nerve stimulation or tcVNS) and (2) delivery to the auricular branch (transcutaneous auricular vagus nerve stimulation or taVNS). Jin and Kong (12) outline key points supporting tVNS application in ASD, namely: (1) tVNS can modulate the core functions impaired in ASD, (2) tVNS can modulate the immune function, where atypical markers are associated with ASD (14), and (3) tVNS may be able treat comorbidities such as epilepsy (15, 16). and depression (17), which are at increased incidence in ASD (14). A noninvasive approach such as tVNS has considerable risk reduction in contrast to invasive VNS. VNS, when delivered invasively (i.e., through an outpatient surgical procedure), is US Food and Drug Administration (FDA) approved for treatment resistant epilepsy and depression. Non-invasive VNS has also recently obtained FDA approval for multiple indications, demonstrating its clinical utility: tcVNS (gammaCore for cluster headache) and taVNS (Sparrow Therapy System for opioid withdrawal). Non-invasive VNS is particularly appealing as it is safe (low-intensity), has no surgical risk, and is low cost. With the recent finding of a beneficial effect of propranolol on anxiety in ASD in a double blind placebo controlled trial (11), this raises the question for the potential of tVNS for the treatment of anxiety by targeting sympathetic/parasympathetic balance in a nonpharmacological manner.

Additionally, taVNS has been shown to enhance recognition of facial emotions (18), of particular importance with the facial attention and emotion recognition impairments widely recognized in ASD (19). Benefits have already been observed, with significant effects on core ASD symptoms in patients receiving VNS for the treatment of co-occurring epilepsy (20). Additionally, given the interrelationship between the autonomic nervous system, anxiety, and gastrointestinal (GI) problems in ASD (21), effects on the GI system might also be anticipated. The GI system is highly interrelated with arousal systems in the body (22), and so approaches targeting the autonomic nervous system (23) may have significant impact on anxiety and GI symptomatology in ASD. Further, GI disturbances are among the most common group of co-occurring conditions in ASD, which are often associated with other behavioral changes, and are frequently refractory to treatment (24).

A number of studies have examined noninvasive brain modulation effects in ASD, including repetitive transcranial magnetic stimulation, transcranial direct current stimulation, and intermittent and continuous theta burst stimulation, reporting cognitive and behavioral effects, including on repetitive behaviors and executive function, in limited studies, and with limited data on long-term outcomes (25–27). To our knowledge, we performed a first study on the potential use of taVNS as a treatment for ASD symptoms, with particular interest in anxiety, focusing on demonstrating feasibility. If successful, this would support for additional investigations, and hopefully ultimately lead to a *non-drug* treatment option with minimal side effects, readily available to broader ASD patient populations. Further, there is substantial interest in exploring protocols that can be delivered at-home. The burden of attending a clinic every day for treatment is impractical due to time constraints (professional and personal) for patients/families and scheduling challenges in the clinic setting coupled with disability that the patient may be managing. Further, the ability to deliver stimulation at-home allows planning larger sample size studies, more sessions, and rapid recruitment, facilitating the opportunity to assess clinical utility of an intervention. This study will also contribute critical preliminary data to assess the safety and utility of home administration, and determine the ability to properly use the device in this setting.

We previously developed and validated a remotely-supervised protocol for a related electrical stimulation modality called transcranial direct current stimulation (tDCS), where participants are able to complete sessions at home (with or without proxy help from family or caregivers) while being supervised by clinical staff (28–30). Translating our experience to tVNS administration, we used a specially designed device and protocol that only authorizes remote stimulation by caregivers after successful demonstration of proxy administration to the supervising clinical staff. Our study reports the details of the first remotely supervised taVNS (RS-taVNS) protocol, feasibility, and initial efficacy outcomes for a treatment of ASD symptoms.

## 2. Methods

### 2.1. Study population

**Figure 1:**
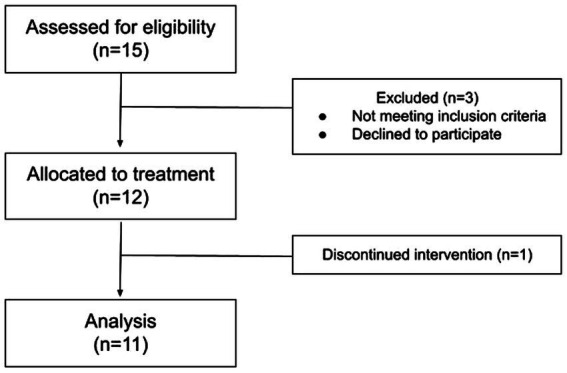
CONSORT flowchart for the single-arm, open-label, pilot study in patients with ASD.

12 participants with ASD (4 female) aged 7–16 (mean 13.1) were recruited from the Thompson Center for Autism and Neurodevelopment (TC) at the University of Missouri – Columbia (MU) (Figure 1). The inclusion criteria were the following: (1) Age between 7–17 years; (2) participants and their parent/guardian must read and write in English; (3) diagnosis of Autism Spectrum Disorder as defined by ADOS-2 (31). and DSM-5 (32). criteria; (4) full-Scale IQ of ≥70; (5) subject must have anxiety, defined as meeting cutoff (total score of 49 or higher) for moderate anxiety on the CASI-R at baseline.

The exclusion criteria were the following: (1) Use of the following medications: sleep medications (melatonin is okay to use), blood pressure medication (propranolol); (2) severe psychiatric diseases (e.g., bipolar, schizophrenia, major depressive disorder); (3) severe neurological illness (e.g., stroke, seizure history, unexplained syncope); (4) bradyarrhythmia; (5) history of head trauma, brain surgery, or tumor; (6) intracranial metal implantation, pacemaker, or other implanted device; (7) history of adverse reaction to electrical nerve stimulation; (8) allergic reaction to adhesives or electrodes; (9) otologic abnormalities that prohibit placement of the device; (10) pregnancy. All female participants of childbearing potential had a urine pregnancy test prior to participation, with a positive result excluding the participant from participation. The study was approved by the University of Missouri IRB and all procedures were completed through the HIPAA- compliant data collection platform ElectraRx (Soterix Medical Inc., New Jersey).

### 2.2. Stimulation system

The taVNS device used in this study is specifically designed to facilitate remote delivery (33). The device, electrodes, and the ElectraRx portal were developed following our extensive experience with home tDCS application and leverages similar concepts. The device (Model 0125, Soterix Medical) includes two modes: administration (admin) and stimulation. The admin mode accessible only by an access code (provided to clinical staff) is used to select stimulation settings and the number of intended sessions. Depending on the number of sessions, unique one-time use “activation codes” are generated. These codes are stored at the clinical center and made available whenever the device needs to be used by the subject/caregiver. Once the stimulation course has been set, the device is handed to the subject. The prescribed stimulation course cannot be altered without the access code. Therefore, any attempts to misuse (i.e., deliver repeated sessions) is simply avoided by dispensing activation codes at a frequency determined by the clinical staff. At home, the subject can only enter one device mode (i.e., stimulation). The device first performs an impedance check ensuring subjects have donned the electrodes correctly and are only allowed to proceed to the next step upon obtaining acceptable contact quality. To help ease of administration, the device displays quality in three levels (“good, “moderate, or “poor”). The subject is prompted for this aforementioned activation code only after obtaining good or moderate contact quality. Upon valid code entry, the device delivers the programmed course. The device only displays the time remaining and the contact quality level throughout the stimulation. If contact quality becomes poor, the device does not “cut-off,” and continues to deliver the maximum possible current intensity constrained by the maximum available voltage limit of the device. Upon detection of poor quality, the device will continuously beep (softly), giving the user 30 s to improve the contact. The device enters into a forced “pause” state if the remedy is not performed in time. This device feature ensures that the device delivers the programmed course to the extent possible. Further, during stimulation, the user may abort the session at any time during stimulation. For post-stimulation assessment, the device logs stimulation history of each session such as number of pause events, critical time, total stimulation time, etc. The period of time under poor contact quality is considered the critical time.

### 2.3. Procedure/study design

Potential participants were required to complete an initial pre-screen for eligibility and provided assent to the procedures with consent being provided by the participant’s parent, legal guardian, or power of attorney. At the initial visit, participants underwent baseline assessments and parents/aide (caregivers) were trained on the use of the stimulator and electrodes. The caregivers were subsequently required to demonstrate successful device operation and electrode placement on the prospective subject before being cleared to participate. At the first visit, participants completed an individualized intensity titration test with the taVNS device. Before stimulation, the electrode placement location was cleaned with an alcohol wipe and lint free pad. The stimulation waveform consisted of monophasic pulses and were delivered at 20 Hz and with 100 μs pulse width, within the range of published stimulus parameters (15, 34–43). Stimulus intensity was determined by ramping the current from 0 to the threshold of discomfort, then reduced to 80% of threshold, as per prior investigations (35–37, 41, 42). Stimulation was delivered through self-adhesive 9 × 12 mm hydrogel stimulation electrodes (RELI-stick, Soterix Medical) placed over the auricular branch of the vagus. Per protocol, the return electrode for taVNS was required to be placed anterior to the tragus to minimize off-target stimulation. Caregivers received a document with important information regarding the study protocol and how to properly use the device in the home. Instructions were given to perform a stimulation session every night until the scheduled visit at the 2 week mark (i.e., for 13–15 nights). The taVNS device was programmed by the clinical staff to deliver active stimulation (i.e., no sham), at personalized stimulus intensity, 20 Hz, 100 μs, 60 min duration for 13–15 sessions. Each caregiver/participant duo received the same study materials to take home that included the aforementioned document, taVNS device, electrodes, battery charger, and alcohol wipes.

Stimulation was administered at home in the evening around the participant’s bedtime with stimulation starting at “lights out.” Delivery during this time period had the advantages of consistency of time and disposition of participants. Electrode application was recorded via photograph of the ear (with face occluded for privacy) and sent to research staff to determine electrode placement accuracy for the first night for all participants. If research staff determined that the photographed placement was inaccurate they provided additional instruction to the parent to correct placement. Caregivers were asked to send a photograph of placement for the second night and thereafter, if caregivers could not demonstrate accurate placement. The device was programmed to deliver the individual’s prescribed current intensity upon entering the activation code provided by the research staff. The code was only provided upon appropriate contact quality reading (i.e., “good” or “moderate”). The device delivered stimulation at the programmed individualized intensity every night. Stimulation could be paused and then resumed. Upon resumption, the device would only deliver stimulation for the remaining time. The ElectraRx online platform was used to monitor codes and session completion. At the end of the 2 week period, participants returned to the clinic for assessment. Participants were compensated $50 for the two in-person visits.

### 2.4. Outcomes

Feasibility of RS-taVNS delivery in this population was our primary outcome measure. Compliance (or stimulation adherence) and safety measures were used to test for feasibility. Compliance was only assessed at the end, and providing feedback was not incorporated in the design. The investigators were blinded to the degree of compliance at the time of assessments. Our secondary outcome measures evaluated initial efficacy reflected by changes from baseline to study end (Table 1). We measured subject compliance as the amount of time that the subject received stimulation out of the total amount of time that the subject was supposed to receive stimulation. 100% compliance required that the subject received stimulation for the entire 60 min session every night that they had the device at home. To identify a study success criterion, we determined the average compliance across recently conducted taVNS trials involving multiple sessions (16, 49–53). We observed a wide completion range (70–100%) and settled on a value midway (85%) considering pediatric patient population, stimulation delivery time (evening before bedtime), and time (60 min sessions).

**Table 1:** Study measures.

	Baseline measures	Daily measures	Endpoint measures
Anxiety	Children’s Anxiety Sensitivity Index (CASI-R) (44, 45) and Parent-Rated Anxiety Scale for ASD (PRAS-ASD) (46)		CASI-R and PRAS-ASD
Sleepiness	Cleveland Adolescent Sleep Questionnaire (CASQ) (47)		CASQ
Anxiety, GI symptoms, Social Interactions, Overall ASD	Clinical Global Impression Severity (CGI-S) (48)		CGI-S and (CGI-I)
Subject compliance and compromised sessions	-	Stimulation contact quality (impedance), stimulation time, critical, and pause events	Stimulation history assessment from device

### 2.5. Secondary outcomes

In addition to determination of feasibility, anxiety assessments were performed in order to collect pilot data on efficacy, while also assessing sleep to determine whether the intervention interfered with sleep.

#### 2.5.1. PRAS-ASD

Anxiety was assessed with a measure that has been developed specifically for use in ASD populations, the Parent-Rated Anxiety Scale for Youth with Autism Spectrum Disorder (PRAS-ASD) (46), developed in part for the purpose of monitoring anxiety response in trials. The scale items were generated from a series of focus groups with parents of children with ASD. The bank of items from the focus groups were included in an online survey and completed by 990 parents. A systematic analysis identified 25 items. The new measure was then validated in a separate clinical sample. The PRAS-ASD, therefore, has demonstrated reliability and validity and consists of 25 questions related to anxiety ranging from 0 (none) to 3 (severe), that can be used to assess severity of anxiety in youth with ASD and evaluate change with treatment.

#### 2.5.2. CGI-S/CGI-I for anxiety

The Clinical Global Impression of Severity (CGI-S) and Clinical Global Impression of Improvement (CGI-I) are semi-structured interviews that have led to reliable and validated primary outcome measures in clinical trials for other cognitive disorders (48). and are currently in wide use in clinical trials in ASD. The CGI-I consists of a 7-point subjective scale assessing change from baseline. On this scale, scores of 1, 2, and 3 represent marked, moderate, and mild improvement, respectively. A score of 4 represents no change. Scores of 5, 6, and 7 represent mild, moderate, and marked worsening, respectively (48). The CGI-I was collected at the final visit. The CGI-S is a similar 7-point subjective scale for severity (score of 1 is no symptoms at all and score of 7 is the most severe symptoms possible), which was assessed at each time point including at the beginning to provide a baseline upon which to base the CGI-I (5 min- parent and clinician). CGI-S and CGI-I scores from both the parent/caregiver and the blinded clinician were utilized. A similar rating was also utilized for gastrointestinal symptomatology as well as for social interaction and overall ASD symptomatology.

#### 2.5.3. CASI-R

To address anxiety for symptoms-based issues, the CASI-R was assessed at baseline and at follow-up. As above, it is a scale of overall and specific types of anxiety among children and adolescents (44, 45). with a focus on anxiety -associated symptoms and fears of these anxiety-associated manifestations. Internal consistency results and test–retest reliability coefficients are excellent. The CASI-R is a well validated instrument that is widely used in ASD in a wide age range.

#### 2.5.4. Sleep assessment

In addition, to assess the effects of tVNS on sleep, the Cleveland Adolescent Sleep Questionnaire (CASQ) was administered at baseline, and at follow-up. The CASQ is a 16-item checklist designed to measure excessive sleepiness among adolescents (47). The student version is to be filled out by adolescents, but can also be completed by a parent or caregiver.

### 2.6. Analysis of secondary outcomes

The secondary outcomes were compared between baseline and follow-up for each of the variables using Student’s *t*-test for an exploratory analysis for generation of pilot data for future studies. All variables were normally distributed. These results are interpreted with caution due to the open-label nature of the study. However, the CGI-S and CGI-I were only reported in a descriptive manner, as these are nonparametric outcomes, not allowing for valid statistical comparison with the methods utilized for the other variables.

## 3. Results

### 3.1. Demographics and clinical features

Subject ages ranged from 7–16 (mean 13.1) and had an anxiety disorder. Individual demographic data, in addition to aspects relevant to level of functioning, are shown in Table 2. For the co-occurring diagnoses among the participants, six participants had been diagnosed with generalized anxiety disorder, four with unspecified anxiety disorder, and one with social anxiety disorder. Mean individually titrated intensity for the 12 subjects was 0.94 ± 0.57 mA. Subject 4 dropped out after 3 nights due to scheduling of “Taekwondo practice in the evening” which precluded continued participation. The subject was in full compliance for the first 3 nights. Therefore, the remaining 11 subjects are included in the formal analysis.

**Table 2:** Demographics and clinical features.

Subject	Age	Male/Female	Anxiety disorder	Full Scale IQ	ADOS comparison score
1	14	Male	Unspecified anxiety disorder	97	10
2	15	Female	Generalized anxiety disorder	102	6
3	11	Male	Unspecified anxiety disorder	108	10
4	14	Male	Generalized anxiety disorder	107	9
5	15	Male	No specified anxiety disorder	71	10
6	16	Female	Generalized anxiety disorder	77	4
7	7	Male	Generalized anxiety disorder	97	7
8	11	Male	Unspecified anxiety disorder	93	10
9	16	Male	Unspecified anxiety disorder	86	8
10	12	Male	Social anxiety disorder	99	7
11	12	Female	Generalized anxiety disorder	95	5
12	14	Female	Generalized anxiety disorder	71	9

Subject 4 (highlighted in gray) dropped out from the study and is not considered in the final analysis.

### 3.2. Primary outcomes

**Figure 2:**
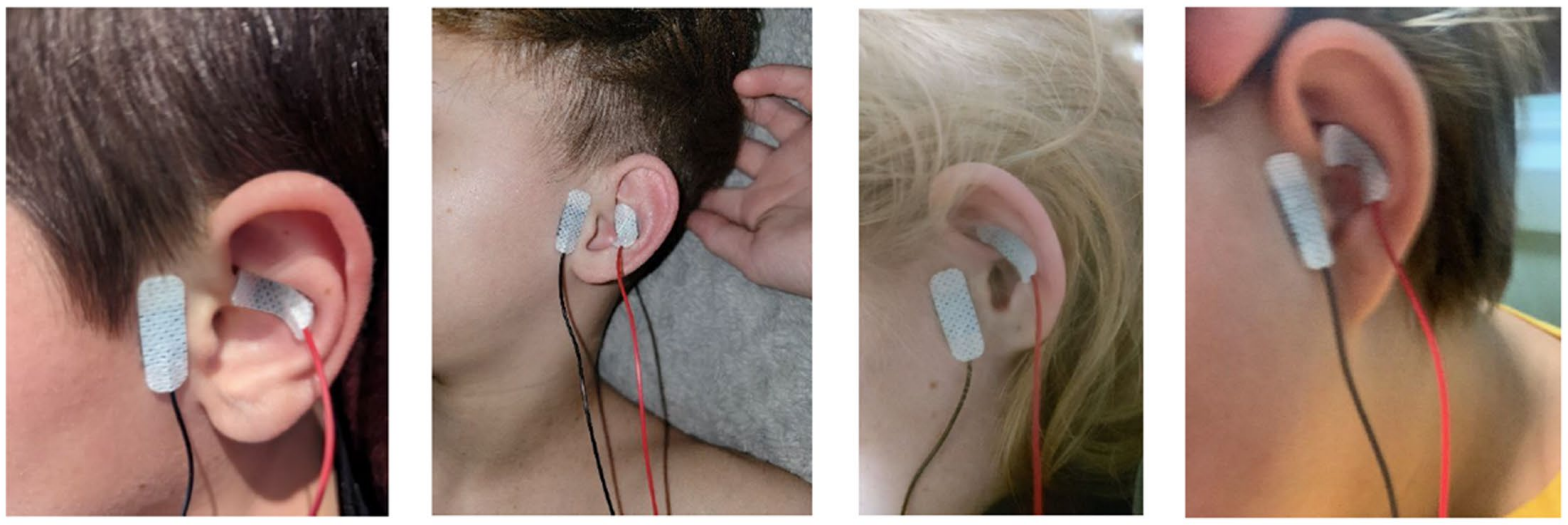
Stimulus location and example application in four randomly selected participants. The first two images from the left are from male participants. The remaining two images are from female participants.

Feasibility: The caregiver-participant combination administered the stimulation 88.5% of the nights (i.e., considering all nights for the full 60 min) they had the “device at home.” A device not at home situation is when the subject would forget the device at the other parent’s house or grandparents’ house. We note that any stimulation outside of a professional healthcare facility is considered as home stimulation, consistent with the regulatory definition (United States FDA). Therefore, the “device not at home” situation here, simply refers to the physical non-availability of the device. The 11.5% out-of-compliance situation stemmed from a combination of both truncated sessions (less than 60 min delivered) and missed sessions. For truncated sessions, completion score of the session was accordingly lowered (e.g., 30 min would score a 0.5 instead of a 1). The reasons for a truncated session reported included lack of time before bed to complete a full session and accidental aborting the session. Since single use device unlock codes were issued at a 24 h interval, a new session could not be delivered on the same day. The reason for a missed session ranged from not feeling well that day, busy schedule, caregiver having a migraine, subject refusing to get stimulation, and device non-availability. Of the 11 subjects, 2 subjects reported not having the device at home on some nights. Photographs of electrode application sent to the research staff for verification purposes are shown in Figure 2.

With respect to contact quality, mean critical time duration of poor contact quality over the entire course of treatments was 190 ± 120 s per subject. This equated to a total of ~3.2 min of stimulation not being delivered at the programmed individualized intensity. Further, we observed a mean stimulation time of 734.9 min (or 12.25 h) for the sample. Therefore, programmed intensity was delivered for ~99.6% of the time. An average of 2.18 ± 2.04 pauses over the series of treatments were recorded for each subject. This reflected robust caregiver-participant capability to correct for atypical contact quality situations and minimizing the need for several forced interruptions.

**Figure 3:**
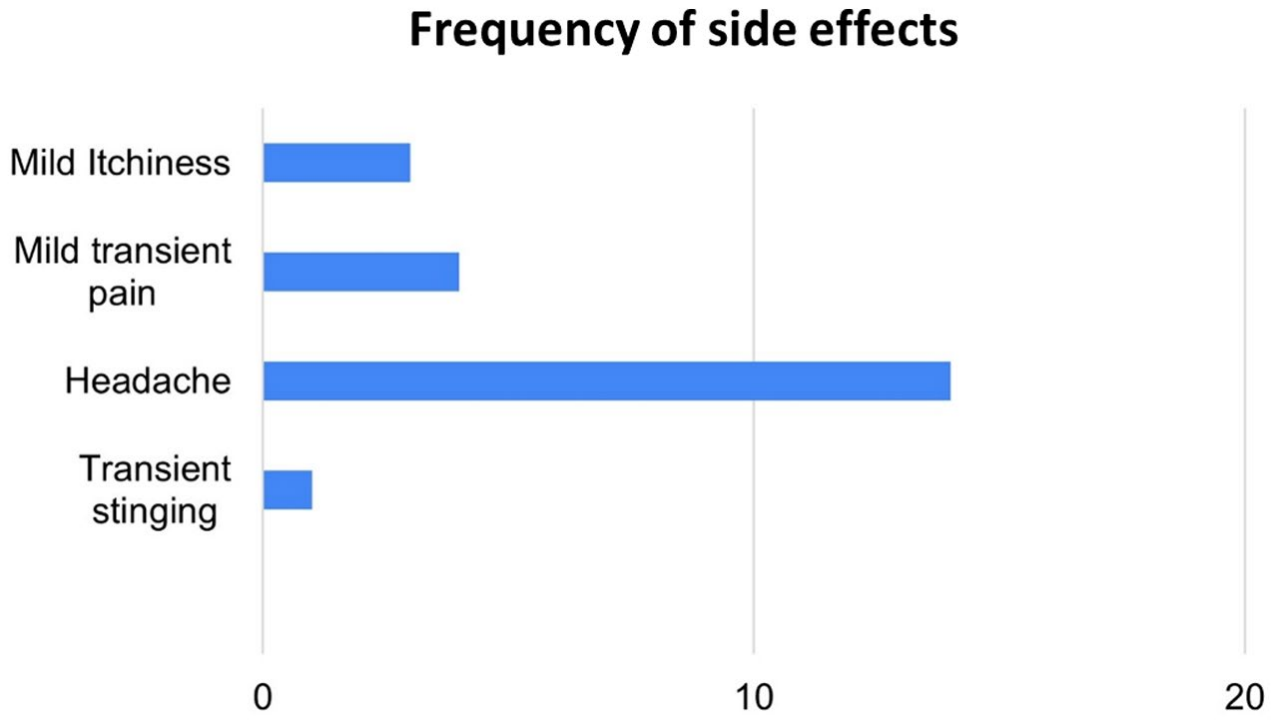
Side effects reported across active taVNS sessions using protocol (*n* = 132).

Only mild expected adverse events were reported by some of the subjects as shown in Figure 3. A total of 22 adverse events were recorded over the course of 132 stimulation sessions. It is to be noted that all reports of headache were by a single subject. The subject received stimulation for intended 14 nights (100% compliant), so the reported headaches did not deter participation. Headache is not considered transient here as the participant reported the headaches to sometimes continue to the next day.

After headache, the next common adverse event was mild transient pain at the electrode sites. This was reported by 3 subjects with each reporting mild pain upon starting stimulation and going away after some time.

### 3.3. Secondary outcomes

**Figure 4:**
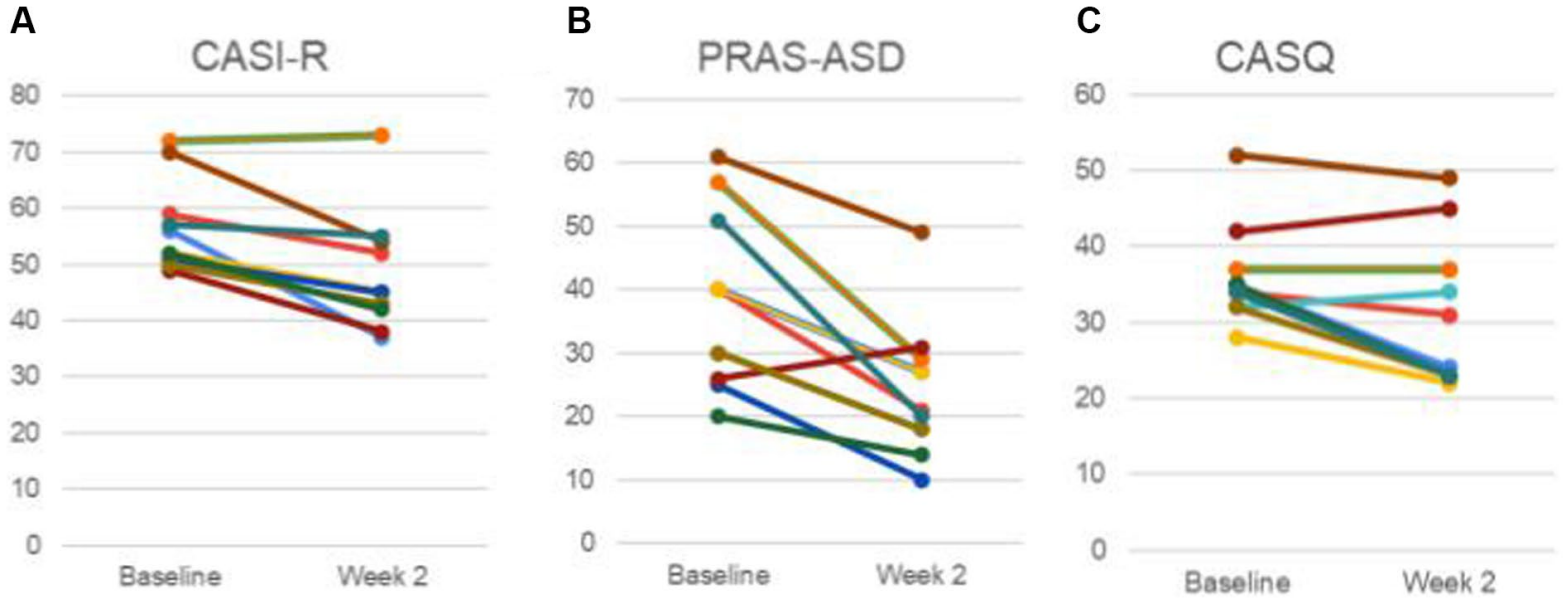
Anxiety and sleepiness Measures. **(A)** Childhood Anxiety Sensitivity Index-Revised (CASI-R). **(B)** Parent-Rated Anxiety Scale for Youth with Autism Spectrum Disorder (PRAS-ASD). **(C)** The Cleveland Adolescent Sleep Questionnaire (CASQ). Data of each participant is represented by a unique color trace. It is to be noted that data for some participants were identical and therefore have overlapping traces.

Anxiety was compared before and after treatment among the 11 participants, with improvements noted (Figure 4). Specifically, average scores on the CASI-R improved from 56.2 (±8.0 sd) to 47.9 (±10.3 sd) (*t*(10) = 4.38, *p* = 0.00069, Cohen’s d = 1.46). Additionally, average scores on the PRAS-ASD improved from 38.2 (±13.6 sd) to 24.0 (±10.5 sd) (*t*(10) = 4.37, *p* = 0.0007, Cohen’s d = 1.46). To assess whether the procedure interfered with sleep, the CASQ was also assessed (Figure 4C). Sleep was not worsened, but rather an improvement was observed, with the CASQ score improving from 36.1 (±6.3 sd) to 31.6 (±9.6 sd) (*t*(10) = 2.22, *p* = 0.025, Cohen’s d = 0.79). Eight of the participants mentioned in their notes falling asleep during stimulation.

**Figure 5:**
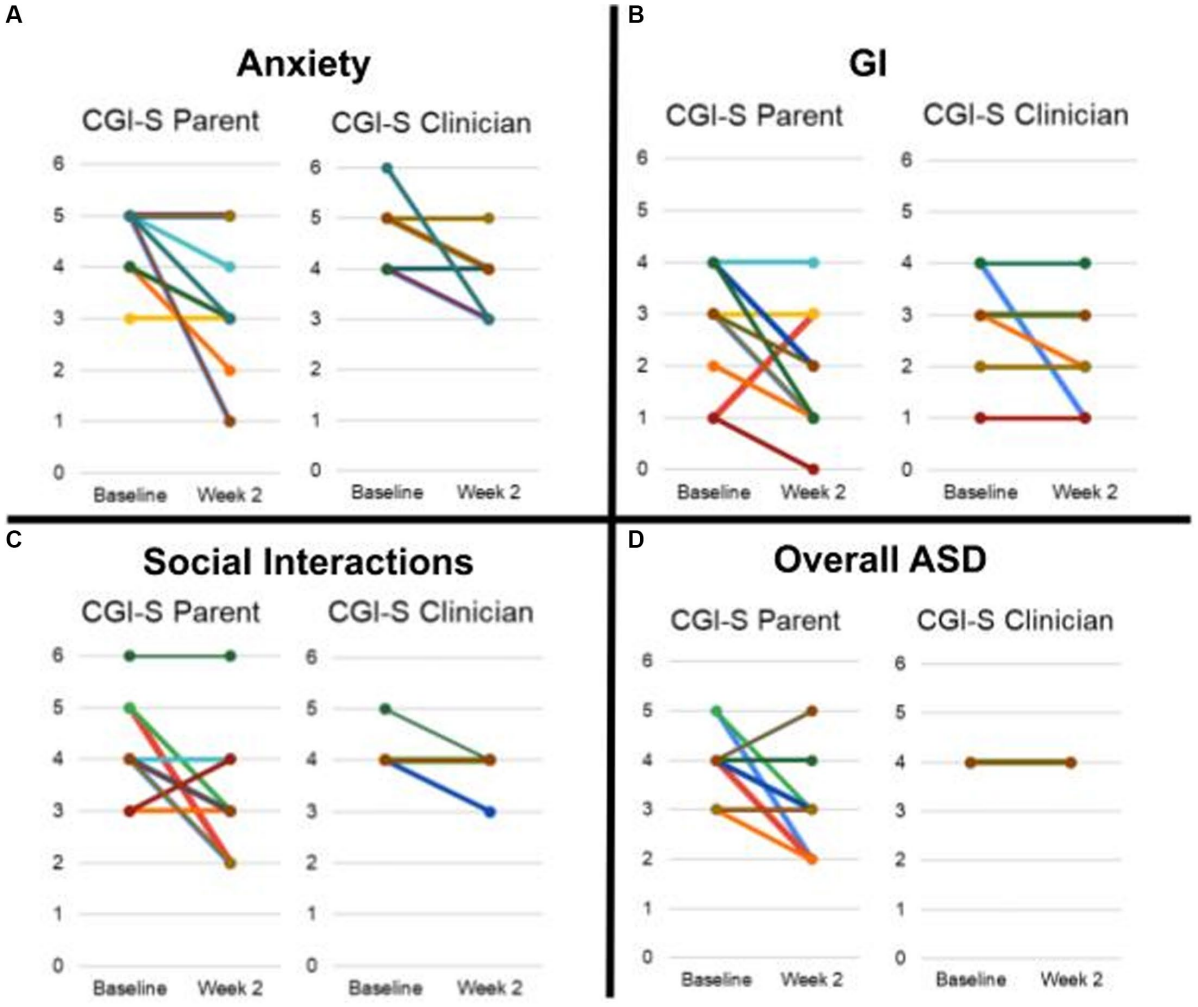
Average CGI-S scores rated by parent and clinician. **(A)** Anxiety. **(B)** GI. **(C)** Social Interaction. **(D)** Overall ASD. Scores of each participant is represented by a unique color trace. It is to be noted that scores for some participants were identical and therefore have overlapping traces.

**Figure 6:**
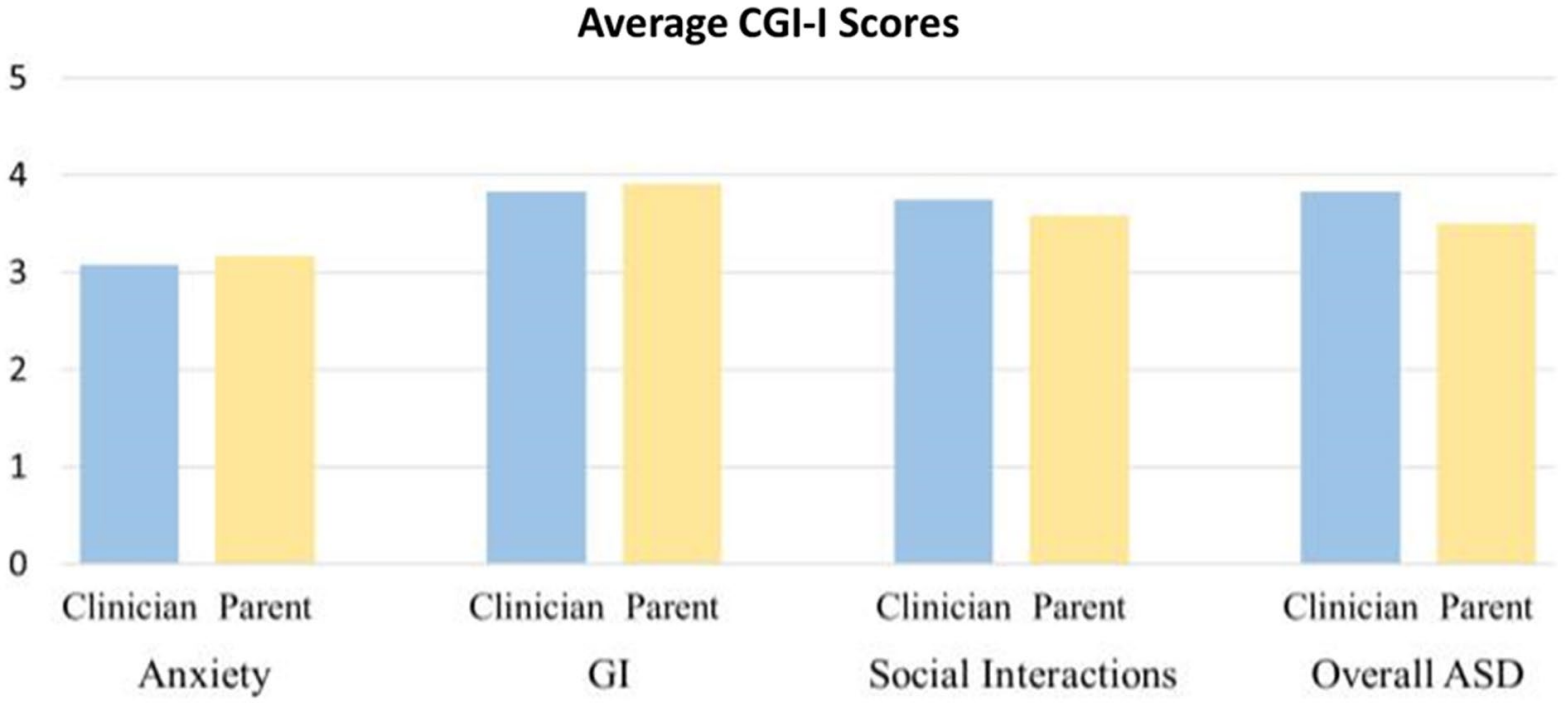
Average CGI-I scores rated by clinician and parent.

CGI-S scores improved for some participants in multiple domains (anxiety, GI, social interactions) in both parent and clinician ratings (Figures 5, 6).

Of the 11 participants, 6 were rated as improved by both the parent and clinician on CGI-I for anxiety, and 3 were rated as markedly improved by the parent and 2 by the clinician for anxiety. For gastrointestinal symptomatology, 2 were rated as improved by the clinician and 1 by the parent on the CGI-I, with none markedly improved.

## 4. Discussion

Our open-label pilot study demonstrated that remote supervised home delivery of taVNS is feasible in patients with ASD. Feasibility was indicated by the high completion rate (~88.5%), tolerability, and robust delivery of the intended stimulation dose. As indicated above, the completion rate is comparable to the reported rate of in-clinic taVNS trials with multiple sessions (16, 49–53). This is especially noteworthy when considering that the subject population had ASD which often causes sensory hypersensitivities that can interfere with compliance (54). The reported side effects across the 132 sessions conducted in the study are in line with the ones previously reported in the literature (55, 56). Post-stimulation assessment performed via retrieving the stimulation history log upon device return verified that the programmed dose (i.e., individualized intensity) was delivered for 99.6% of the time.

The average anxiety (CASI, PRAS-ASD, CGI) and sleep (CASQ) scores were all improved at the 2 week time point. The average PRAS-ASD scores demonstrated a ~ 37% improvement from the baseline scores. Given the small sample size (with main goal to establish feasibility), we are unable to assess efficacy systematically, particularly with the significant limitation of the open label design. However, we note promising initial evidence suggestive of efficacy.

As noted before, our procedures for remote-supervision primarily relied on the device design to prevent unauthorized changing of set intensity and duration, forced correction of poor impedance condition before proceeding with the session, and unlock code dispense at a 24 h interval via the portal. Our trial did not include real-time monitoring through videoconferencing (as employed in some tDCS trials) given the unique requirement to initiate stimulation in the evening around the participant’s bedtime. While remote-supervised trials in tDCS have included a range of video monitoring strategies (for all sessions for entire duration, first few sessions for entire duration, first few minutes for every session, to none), our successful delivery of stimulation relying primarily on combination of caregiver’s direction and device functionality, demonstrates that planning trials is possible in this challenging population.

We note that the ability to offer trial participation to this population who otherwise could not have traveled to the clinic for daily sessions, now allows us to include a wide range of the ASD community for future larger trials. While our study did not specifically include a satisfaction survey, several caregivers provided positive feedback at the final clinic visit. We were also able to recruit participants for this study relatively easily and quickly as opposed to our on-going in-clinic trials at that time, involving the same population. Crucially, the success of our study now facilitates the planning of future studies with more sessions, involving more subjects, which hopefully will help in developing the clinical utility of the intervention. Additional benefits include cost saving in terms of clinical staff and allocation resulting in a more economical therapeutic option.

There exist studies that report successful delivery of taVNS at-home for sessions spanning months (50, 53). We however, note important differences with our study. In Stavrakis et al., adult patients with paroxysmal atrial fibrillation (AF) self-administered taVNS for 1 h daily for 6 months. Patients could change amplitude settings and were required to maintain a daily log with time, application duration, and the amplitude used. Ear-clip style electrodes were used which are easier to self-administer than the one used here. Patients were simply told to hold electrodes either on the tragus (for active stimulation) and the ear lobe (for sham stimulation). The authors did not foresee potential device mis-use and break of treatment allocation blinding (e.g., patient determining on their own that the ear lobe is typically used for the sham arm)- which is ultimately justified given the success of the trial. In Wang et al., adults with mild cognitive impairment (MCI) self-administered taVNS for 1 h daily (over two 30 min sessions) for 6 months (53). While not explicitly stated, it seems that patients could change amplitude settings. Patients were required to maintain a dairy and required to provide responses via telephone or WeChat as needed. The investigators connected with the patient once a week over video to ensure the subject was continuing to stimulate the intended auricular points. The definition of compliance/adherence has varied across these trials. Considering adherence (<=4 missed sessions per month), ~80% were reported to be in compliance for the AF study (50). With “discontinued intervention” being treated as non-compliance for the MCI study, 86.7% subjects were included in the final data analysis (53). While compliance comparisons to our trial are not applicable, we demonstrate that high compliance can also be achieved in patients with ASD and possibly for longer durations – given the positive feedback provided by caregivers.

To our knowledge, our study is the first to demonstrate successful delivery of taVNS at-home in patients with ASD, and in any pediatric population. This technique has advantages over other noninvasive neurostimulation approaches (25–27) due to the more readily accessible technology and lower cost, as well as ease of at-home administration associated with taVNS, in addition to the specificity of targeting to anxiety, as is proposed as the primary outcome for future work, since treatment options for anxiety in the setting of ASD have limited evidence at present. Further, device and protocol design ensure a “higher level of control” than prior at-home trials (i.e., no stimulation setting could be altered or exceed the one session/day regimen). Ultimately for future trials, the patient population and related trial design/allowed deviation will dictate the “level of control” necessary.

There are significant limitations that should suggest caution in the interpretation of any findings reported herein. First, the sample size is quite small, but even more critically, as a feasibility study, this was performed in an open label manner. Any outcomes beyond the domain of feasibility should be taken with extreme caution as they could all be markedly impacted by a placebo effect, which has been observed to be a significant issue in ASD studies (11). Additionally, the parameters selected for study herein were based on experience with other populations, and might need adjustment for optimization in ASD.

Given the recent report of effects of propranolol on anxiety in ASD (11), and this preliminary data targeting the sympathetic/parasympathetic system in a nonpharmacological manner, we would anticipate effects on anxiety with a larger clinical trial in ASD. Therefore, this was the target population for the present study, and we did not examine this intervention in a control group. The ultimate goal will be bringing a nonpharmacological therapeutic option for anxiety in ASD in clinical use, if successful. Initial findings are promising for such an effect, but extreme caution is urged on deriving any conclusion due to the open-label nature of this feasibility trial. A larger double-blinded placebo controlled trial would be needed to begin to establish efficacy. Anxiety would be the likely primary outcome measure based on the findings herein and the findings of other approaches targeting sympathetic/parasympathetic balance (11). However, it would be critical to monitor for effects on other outcomes as well. Follow-up trials should monitor biomarkers to predict best responders (14), and monitor effects related to state vs. trait anxiety, and the impact of stress exposures during the study. However, it is notable that the intervention appears to be well tolerated by most participants, which is promising for the implementation of a subsequent trial.

## Data availability statement

The original contributions presented in the study are included in the article/supplementary material, further inquiries can be directed to the corresponding author.

## Ethics statement

The studies involving humans were approved by the University of Missouri IRB. The studies were conducted in accordance with the local legislation and institutional requirements. Written informed consent for participation in this study was provided by the participants’ legal guardians/next of kin.

## Author contributions

BB, DB, AD, and EP designed the study with important intellectual input from BF. SH, HC, NT, and RD conducted the study including patient recruitment and data collection. BB, DB, AD, and YV completed the data analyses and drafted the initial manuscript. All authors contributed to the final manuscript text, and approved the submitted version.
